# Does perioperative systemic therapy represent the optimal therapeutic paradigm in organ-confined, muscle-invasive urothelial carcinoma?

**DOI:** 10.2144/fsoa-2021-0092

**Published:** 2021-10-28

**Authors:** Luca Scafuri, Antonella Sciarra, Felice Crocetto, Matteo Ferro, Carlo Buonerba, Francesco Ugliano, Germano Guerra, Roberto Sanseverino, Giuseppe Di Lorenzo

**Affiliations:** 1 Oncology Unit, Hospital ‘Andrea Tortora,’ ASL Salerno, Pagani, 84016, Italy; 2 Associazione O.R.A., Somma Vesuviana, Naples, Italy; 3 Department of Experimental Medicine, University of Campania ‘Luigi Vanvitelli,’ Naples, NA, 80131, Italy; 4 Department of Neurosciences, Reproductive Sciences & Odontostomatology, Federico II University, Naples, 80131, Italy; 5 Division of Urology, European Institute of Oncology (IEO), IRCCS, Milan, 20099, Italy; 6 Department of Medicine & Health Science, University of Molise, Campobasso, 86100, Italy; 7 Department of Urology, Umberto I Hospital, ASL Salerno, Nocera Inferiore, 84014, Italy

**Keywords:** adjuvant therapy, bladder cancer, neoadjuvant therapy

Although urothelial carcinoma can occur in any part of the urinary tract, 90–95% of urothelial malignancies arise in the bladder [1]. Conversely, approximately 90% of bladder malignancies have a urothelial origin [2]. According to GLOBOCAN, approximately 570,000 individuals were diagnosed with bladder cancer (BCa) in 2020 worldwide, with approximately 210,000 dying of the disease [3]. Southern Europe is the world area presenting with the highest incidence of BCa in males [2], with peaks of over 70 cases per 100,000 males in certain areas of southern Italy [4]. Cystectomy [5] and radical nephroureterectomy [6] represent the mainstay of treatment of localized urothelial cancer that is not amenable to conservative treatment such as intravescical BCG [7]. While both chemotherapy based on platinum agents and immune therapy based on anti PD-1/PDL-1 agents (programmed death-1/programmed death ligand-1) such as pembrolizumab and avelumab have been definitively proven to extend survival in patients with advanced/metastatic disease [8–10], their effect in terms of survival prolongation in the perioperative setting requires to be better investigated. In a meta-analysis [11] assessing overall survival in 2890 BCa patients who received neoadjuvant platinum-based chemotherapy followed by radical cystectomy and 10,418 BCa patients who underwent radical cystectomy alone, neoadjuvant chemotherapy was associated with a 18% reduction of the risk of death with a hazard radio (HR) of 0.82 (95% CI: 0.71–0.95; p = 0.009). Conversely, in the POUT randomized-controlled trial enrolling an overall sample of 261 patients with urothelial carcinoma of the upper urinary tract staged as either pT2-T4 pN0-N3 M0 or pTany N1–3 M0 after nephroureterectomy, adjuvant chemotherapy based on cisplatin/carboplatin plus gemcitabine compared with observation was associated with a significant 55% reduction of the risk of disease recurrence [12]. Although the POUT investigators concluded that either cisplatin or carboplatin may be used in the adjuvant setting, therapy based on cisplatin versus carboplatin was associated with a HR of 0.35 (p < 0.001) versus 0.66 (p = 0.21), respectively, so we believe that evidence from the POUT trial supports the use of cisplatin as opposed to carboplatin whenever cisplatin is feasible. In this regard, we hypothesize that the use of sequential neoadjuvant plus (in select patients) adjuvant platinum-based therapy may be advantageous compared with adjuvant therapy alone in patients with upper tract urothelial carcinoma, as a substantial proportion of patients are expected to become cisplatin-ineligible after nephroureterectomy.

Sequential use of neoadjuvant and adjuvant therapy in urothelial carcinoma patients may represent a disease-specific paradigm that may be appliable with success both to chemotherapy and immunotherapy. In this regard, we read with interest the results obtained in one recently published meta-analysis that assessed survival differences in patients with pT3-T4 and/or pN+ muscle-invasive BCa who underwent neoadjuvant chemotherapy + surgery with versus without adjuvant chemotherapy [13]. In the overall sample of 3096 participants, of whom 2355 (76.1%) and 741 (23.9%) did not and did receive adjuvant chemotherapy, respectively, adjuvant chemotherapy was associated with a significant advantage in overall survival (HR: 0.84; 95%CI: 0.75–0.94; p = 0.002) and disease-specific survival (HR: 0.56: 95% CI: 0.32–0.99; p = 0.05). Interestingly, the magnitude of the overall versus disease-specific survival advantage may be more limited, possibly due to the medium-to-long term effects of chemotherapy on noncancer-specific survival, which is consistent with findings obtained by our research group, suggesting a higher noncancer mortality in BCa patients undergoing four versus three cycles of neoadjuvant platinum-based chemotherapy [14]. In this regard, perioperative immunotherapy may be more advantageous compared with chemotherapy in terms of noncancer-specific survival. Results from Phase III Checkmate 274 trial conducted in patients with urothelial cancer who were randomized to 1 year anti-PD-1 agent nivolumab or placebo administered after radical cystectomy or (nephro)-ureterectomy have been published recently [15]. Adjuvant nivolumab compared with placebo for a year was associated with a significant HR for recurrence or death of 0.70 (98% CI: 0.55–0.90; p < 0.001), with median disease-free survival (DFS) of 20.8 versus 10.8 months, respectively. Of note, there was a signal of a potential greater effectiveness of nivolumab in patients who had undergone prior neoadjuvant cisplatin-based chemotherapy (HR for progression = 0.51). This finding is consistent with differences in median DFS reported in the Phase III IMvigor010 trial assessing adjuvant atezolizumab versus observation in urothelial carcinoma patients after radical surgery [16], with a median DFS of 16.8 versus 19.4 months in those who had not received neoadjuvant chemotherapy compared with 19.8 versus 16.5 months in those who had received neoadjuvant chemotherapy. We also note that neoadjuvant immunotherapy based on anti-PD-1 agents prior to cystectomy has yielded exciting results. The updated results obtained in the PURE-01 in 114 BCa patients receiving pembrolizumab prior to cystectomy shoed a pT0 rate of 37% (95% CI: 28–46) and a pT ≤ 1 rate of 55% (95% CI: 46–65) [17].

Given the results reviewed here, we hypothesize that the best possibilities of cure in patients with muscle-invasive urothelial cancer are provided by perioperative cisplatin-based chemotherapy + immunotherapy combination. Given medium-to-long term toxicity associated chemotherapy, novel trials may be designed to explore the approach based on delivering a minimum number of cisplatin-based chemotherapy cycles (e.g., three) prior to surgery in combination with immunotherapy and administer immunotherapy only as adjuvant therapy in those who have achieved a complete pathologic response. The perioperative use of pembrolizumab in combination with chemotherapy in cisplatin-eligible muscle-invasive BCa and of pembrolizumab with or without enfortumab vedotin in cisplatin-ineligible muscle-invasive BCa is currently being explored in ongoing Phase III trials [18]. The results of these trials are eagerly awaited and may confirm that the ‘perioperative paradigm’ represents the best therapeutic approach in patients with muscle-invasive urothelial carcinoma undergoing radical surgery.
